# Influence of Fermentation Container Type on Chemical and Microbiological Parameters of Spontaneously Fermented Cow and Goat Milk

**DOI:** 10.3390/foods12091836

**Published:** 2023-04-28

**Authors:** Wafa Mkadem, Valentina Indio, Khaoula Belguith, Olfa Oussaief, Federica Savini, Federica Giacometti, Halima El Hatmi, Andrea Serraino, Alessandra De Cesare, Nourhene Boudhrioua

**Affiliations:** 1 Laboratory of Physiopathology, Alimentation and Biomolecules (LR17ES03), Higher Institute of Biotechnology Sidi Thabet, University of Manouba, BP-66, Ariana 2020, Tunisia; mkademwafa@gmail.com (W.M.);; 2 Department of Veterinary Medical Sciences, Alma Mater Studiorum-University of Bologna, Via Tolara di Sopra 50, 40064 Ozzano dell’Emilia, Italy; 3 Livestock and Wildlife Laboratory, Arid Lands Institute of Medenine, University of Gabes, Medenine 4119, Tunisia; 4 Food Department, Higher Institute of Applied Biology of Medenine, University of Gabes, Medenine 4119, Tunisia

**Keywords:** goat milk, cow milk, spontaneous fermentation, microbiota, fatty acids, phenolic acids, physicochemical properties

## Abstract

Fermented goat milk is an artisanal beverage with excellent nutritional properties. There are limited data on its physicochemical properties, fatty acids, phenolic acids, and on any insight on microbiota. The aim of this research was to conduct a pilot study to compare these parameters in raw cow and goat milk before and after spontaneous fermentation in a clay pot and glass container at 37 °C for 24 h. Both types of milk and fermentation containers significantly affected the pH, acidity, proximate composition, viscosity, and whiteness index of fermented milks. A total of 17 fatty acids were identified in fermented milks, where palmitic, stearic, and myristic were the main saturated acids, and oleic and linoleic acids were the main unsaturated ones. These profiles were primarily influenced by the type of raw milk used. Three to five phenolic acids were identified in fermented milks, where quinic acid was the major phenolic compound, and salviolinic acid was identified only in raw goat milk. Preliminary metataxonomic sequencing analysis showed that the genera *Escherichia* spp. and *Streptococcus* spp. were part of the microbiota of both fermented milks, with the first genus being the most abundant in fermented goat milk, and *Streptococcus* in cow’s milk. Moreover, *Escherichia* abundance was negatively correlated with the abundance of many genera, including *Lactobacillus*. Overall, the results of this pilot study showed significant variations between the physicochemical properties, the fatty and phenolic acids, and the microbial communities of goat and cow fermented milk, showing the opportunity to further investigate the tested parameters in fermented goat milk to promote its production.

## 1. Introduction

Traditional fermented dairy products are essential for the human diet in North Africa and Mediterranean regions. Spontaneously fermented dairy products contain essential nutrients such as vitamins, minerals, proteins, bioactive peptides, fatty acids, and beneficial bacteria that ensure a stable gut microbiota [1]. The comparative composition of goat and cow milk have been previously reviewed in other studies [2,3]. When compared to cow milk, goat milk has a higher β-casein to α_s_-casein ratio, more short-chain fatty acid, medium-chain triacylglycerols (TAGs), and a higher oligosaccharide content [4]. The nutritional composition of goat milk is considered similar to that of human milk, with therapeutic values including digestibility, anticarcinogenic, antimicrobial, and immune boosting properties [5].

During the spontaneous fermentation of milk, indigenous microbiota—mainly lactic acid bacteria (LAB)—and those from the surrounding production environment enter into competition and symbiotic interactions to grow, survive, and even become dominant in the final dairy products, contributing to their composition, texture, appearance, taste, and flavor attributes [6,7]. Traditional fermented dairy products are biologically and biochemically dynamic matrices that are affected by milk type and processing conditions, according to know-how and specific gastronomic heritage, leading to the regional typicity of the developed product [8,9]. Thus, it has been proven in previous studies that both the vessels used for milk processing and fermentation temperature contribute to microbial and nutritional specificities of the final products [10,11]. Traditional handling practices of milk and milk products are based on the use of containers made from locally available materials, including skin bags, calabash from gourds, and clay pot.

Clay containers have been used to ferment cow milk in African countries and are still used in rural areas to produce products with a sour taste, an acidic flavor, and a thicker texture than yogurt [11,12]. Fermentation vessels made with clay pot have been proven to contribute better to flavor development than other materials [13]. Unpasteurized fresh raw milk was left in clay containers to obtain a typical product with an unsweetened taste and smooth texture. Furthermore, fermented dairy products contain functional compounds, including vitamins, fatty acids, and oligosaccharides [14]. Milk fermentation involves the growth of mixed microorganisms. Some bacteria participate in symbiotic interactions, whereas others have altered dominance during fermentation [15,16]. The most common microorganisms involved in fermented dairy products are lactic acid bacteria and yeast. These microorganisms contribute to the technological process and production of metabolites such as enzymes, micronutrients, and acids [17]. However, the use of unsterilized traditional containers to ferment or store milk greatly affects the shelf life of dairy products and the load of pathogenic bacteria in samples that are potentially hazardous to consumer health [18].

Studies describing the physiochemical properties, micronutrient profiles, and the microbial diversity of fermented milk and their variations according to the type of milk and container used for fermentation are limited. Clay pots provide the widest variety of yeasts and lactic bacteria, making them the best option for producing traditional fermented milk [19].

Therefore, this study aimed to investigate the physicochemical properties, fatty acids, and phenolic acids of fermented cow and goat milk in glass and clay containers. Microbial community composition of raw and fermented milks was also assessed using 16S metataxonomic sequencing analysis.

## 2. Materials and Methods

### 2.1. Raw Milk Samples and Fermented Milk Preparation

Raw cow milk samples were collected from a local producer (Medenine, Tunisia), at a 10-min walk distance to the laboratory, transported in sterile bottles, stored at 5 °C, and immediately transferred for analysis and processing. Raw goat milk samples were collected from an experimental farm belonging to the Arid Land Institute (IRA Medenine, Tunisia).

Clay containers made from dried clay with a volume capacity of 250 mL were used (Graphical abstract). Glass containers having the same volume capacity were also used. The clay pots were pre-washed under running hot water (60 °C/5 min) while the glass containers were sterilized before experimentation.

For each type of milk, two different fermentation vessels were used: clay pots and glass containers. Spontaneous fermented goat and cow milk were prepared in each container and were monitored simultaneously at 37 °C in a laboratory incubator (Labtech Daihan, Labtech, Seoul, Republic of Korea). The same starting time of fermentation was applied until milk coagulation (a final pH value of ~4.6–4.4 was achieved between 14 and 20 h for different samples). Milk samples (~20 mL) were aseptically withdrawn during fermentation at different intervals for analysis. Two batches were performed for goat or cow milk fermentation in different.All following physicochemical and microbial analyses were conducted in triplicate.

### 2.2. Acidification and Lactic Acid Bacteria Growth Kinetics

The pH and titratable acidity of milk samples were determined at intervals of 2 h during the 24 h of fermentation. pH measurement was performed using a pH meter (Jenway, Staffordshire, United Kingdom) after calibration by pH 7.0 and 4.0 standard buffer solutions before each test with a selected resolution of 0.01 pH and an accuracy of ±0.003 pH. Acidity was conducted by NaOH titration. Briefly, 10 mL of milk sample was added to the phenolphthalein solution, and the mixture was titrated by NaOH 0.1 N until the color changed to pink for 30 s. Lactic acid was expressed as g lactic acid/L [20]. LAB were enumerated on MRS agar (Biolife Italiana Srl, Milan, Italy) every 3 h, according to the official methods of ISO 7889:2003 [21].

### 2.3. Proximate Composition and Apparent Viscosity

Fermented milk samples were analyzed for proximate composition. Protein content was determined according to the Kjeldahl method and calculated by multiplying N (the total nitrogen) with the conversion factor 6.38. The total solids content was determined by drying the samples at 105 °C in a hot air oven (Memmert, Germany) until a constant weight was achieved. The total ash was determined using a furnace (Nabertherm controller B 170, Nabertherm GmbH, Lilienthal, Germany) at 600 °C for 6 h. The total fat was determined by the Gerber method using a Gerber Centrifuge (Funke Dr. N. Gerber, Berlin, Germany), and lactose was measured by Lactoscan MCCW (Milkotronic Ltd., Nova Zagora, Bulgaria) [22].

The water activity of the fermented milk samples was determined using a LabMaster-aw neo water activity meter (Novasina AG, Lachen, Switzerland), and the apparent viscosity was evaluated using a Brookfield viscometer (DV-E, Brookfield Engineering Laboratories, Middleborough, MA, USA) [23].

### 2.4. Whiteness Index (WI)

The color parameters CIE L* a* b* of the fermented milk samples were determined using a Chroma Meter CR-400/410 (Konica Minolta, Osaka, Japan) and were used to calculate the whiteness index (WI) based on Equation (1) according to Kamal-Eldin et al. [24].

WI = 100 − ((100 − L*)^2^ + a*^2^ + b*^2^)^1/2^ (1)

where L* is lightness to darkness (100 to 0), a* is reddish (+)/greenish (−), and b* is yellowish (+)/bluish (−) color components.

### 2.5. Fatty Acid Methyl Esters (FAME) Analysis

Fat fractions of the raw and fermented milk samples obtained by centrifugation (5000× *g*, 20 min, 4 °C) using a Sorvall LYNX 6000 centrifuge (Thermo Fisher, Waltham, MA, USA) were dissolved in a methanolic potassium hydroxide solution (KOH, 2 mol/L) and mixed thoroughly with hexane to extract fatty acid methyl esters (FAMEs). FAMEs were analyzed using a gas chromatograph (Shimadzu model QP2010; Shimadzu, Tokyo, Japan) coupled with a mass spectrometry detector. The fatty acid composition was determined by comparing the obtained peaks to the chemical standards of the GC-MS and Wiley 275 mass spectra library database (Software, D.03.00, Palo Alto, CA, USA).

### 2.6. Phenolic Compounds Analysis by LC-ESI-MS

Milk extracts were prepared according to Vázquez et al. [25], and were subjected to quantitative phenolic compounds analysis using an LC-MS-2020 quadrupole mass spectrometer (Shimadzu, Kyoto, Japan) equipped with an electrospray ionization source (ESI) in the negative mode, as previously described by Jrad et al. [26]. The mass spectrometer was set to the negative ion mode, with a capillary voltage of 3.5 V, nebulizer gas flow of 1.5 L/min, dry gas flow of 12 L/min, and DL temperature (dissolution line) of 250 °C. The temperature of the block source was 400 °C, the voltage detector was 1.2 V, and the complete scan spectra ranged from 50 to 2000 *m*/*z* (Bedford, MA, USA). The phenolic compounds were identified by matching the obtained retention times and mass spectra to chemical standards of >98% purity obtained from Sigma Chemical Co. (St Louis, MO, USA).

### 2.7. Statistical Analysis

Results were expressed as the mean ± standard deviation (SD). A two-way analysis of variance (ANOVA) was conducted based on the presence of two factors: type of milk, container used for milk fermentation, and interaction level. The type III sum of squares described the significance of each factor. Tukey’s post hoc test was used to analyze differences with a 0.05 significance level. Statistical analysis was performed using XLSTA software version 2019 (Addinsoft, Paris, France).

### 2.8. Microbial Community Composition

#### 2.8.1. DNA Extraction

The genomic DNA of raw cow and goat milk and the corresponding fermented milk (after 24 h) in the glass container and clay pot were extracted using the DNeasy Power Food Microbial Kit (Qiagen, Hilden, Germany), as previously described [27]. Additionally, a sample of an artisanal commercial fermented cow milk “Lben” from a local producer was also analyzed as a control for microbial diversity analysis. Briefly, 50 mL of samples were centrifuged at 4300× *g* for 20 min at 4 °C. Then, 1 g of pellet was centrifuged at 13,000× *g* for 1 min at room temperature. The supernatant was discarded, and the 250 mg of the second pellet were used for DNA extraction.

#### 2.8.2. NGS, Bioinformatic and Statistical Analysis

The Illumina 16S Library preparation protocol was adopted to generate the libraries by amplifying the V3 and V4 regions of the 16S rRNA. Sequencing was performed on the Illumina MiSeq platform using the MiSeq Reagent kit v2 500 cycles.

The bioinformatic analysis was performed using a pipeline based on QIIME2 (http://qiime.org/ (accessed on 13th January 2022)). First, the dada2 algorithm was applied in order to denoise, merge forward, and reverse sequences per each pair. Then, the taxonomic classification was performed by applying the Sklearn classifier implemented in QIIME and adopting the Greengenes 13_8 97% OTU dataset as a reference.

## 3. Results

### 3.1. Effect of the Fermentation Container on Acidification and Lactic Acid Bacteria Growth

**Figure 1:**
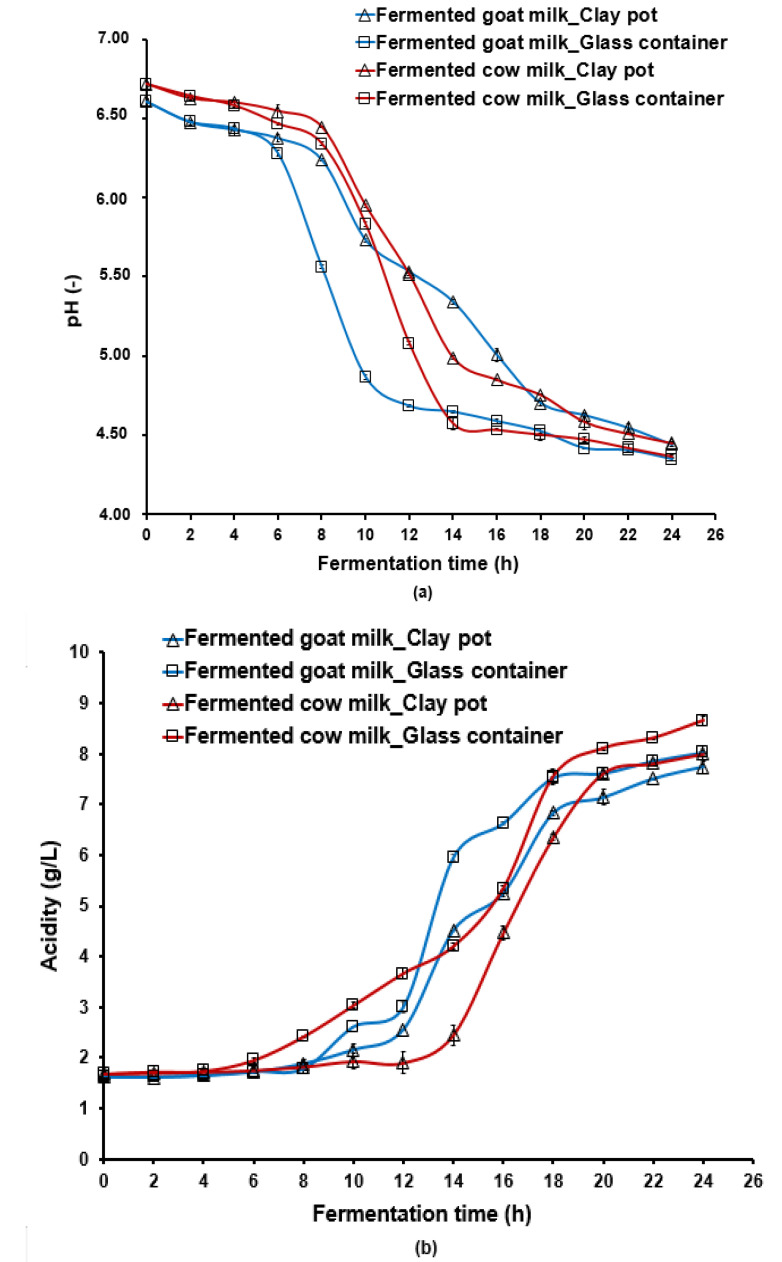

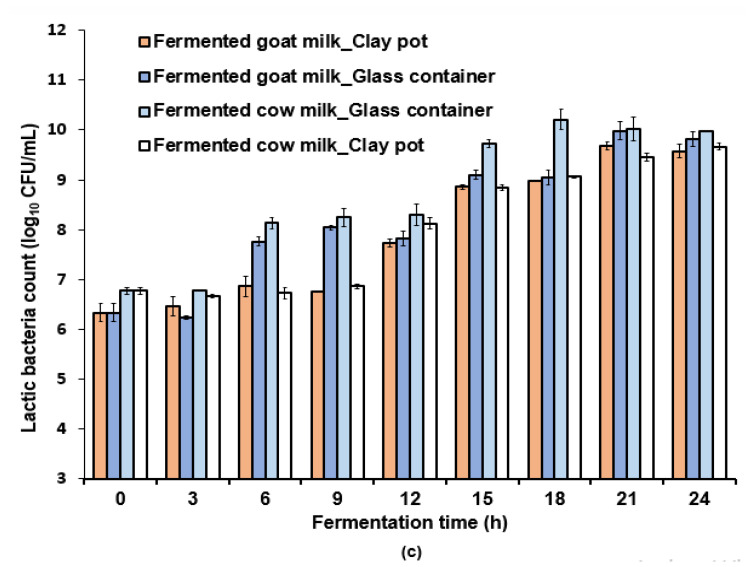
pH (**a**), acidity (**b**), and total lactic acid bacteria count (**c**) changes during cow and goat milk fermentation at 37 °C during 24 h using clay pots or glass containers. Groupwise summary statistics result for pH, acidity, and lactic bacteria count for the different fermentation times were given in Supplementary Tables S1 and S2.

Changes in pH and acidity are shown in Figure 1a and in Figure 1b. During fermentation, milk acidification kinetics in the clay pot and glass container were statistically different (*p* < 0.05). The pH ranged from 6.61 ± 0.02 to 4.44 ± 0.01 for goat milk using clay pot and from 6.61 ± 0.02 to 4.35 ± 0.01 during fermentation in glass container. For cow milk, pH varied from 6.72 ± 0.02 to 4.45 ± 0.01 for fermentation in clay pot and from 6.72 ± 0.02 to 4.37 ± 0.01 for fermentation in glass container (Figure 1a). The use of a glass container for milk fermentation decreased the pH below compared to that achieved using a clay pot (Supplementary Table S1). Furthermore, milk acidification rate was greater when using a glass container for cow and goat milk (Figure 1b).

Statistical analysis based on the type III sum of squares applied to the explanatory variables: type of milk, the container used, and their interaction term showed that all variable terms had a significant effect on pH and acidity variations depending on the fermentation time (Supplementary Table S1). In the first 6 h of milk fermentation, the term type of milk is significant (*p* < 0.05) for pH and acidity. Afterwards, a significant effect was also found for container type and interaction term (container × milk). Both milk type and container significantly influenced the pH and variation between samples within 6 and 24 h of milk fermentation. The mutual interaction term “type of milk × container” was found significant (*p* < 0.05) for all measurements from 8 h to 22 h (Supplementary Table S1).

The total LAB count increased slightly during milk fermentation (Figure 1c). During the first 3 h of fermentation, the variation in LAB count was attributed to the difference in the type of milk (Supplementary Table S2). Initial LAB count for cow milk was slightly higher (6.8 log_10_ CFU/mL) compared to that of goat milk (6.3 log_10_ CFU/mL). From 6 to 9 h of fermentation, a significant effect of the container was noted; cow milk in a glass container recorded a maximal LAB count of approximately 8.1–8.2 log_10_ CFU/mL. At 12 h of fermentation, only the variable type of milk explained the significant difference among different samples (*p* < 0.05) (Supplementary Table S2).

From 15 to 18 h, both factors and the interaction type of milk *×* container significantly affected LAB count, and after that, only the variable container explained the significant difference (Supplementary Table S2). At 24 h, fermented milk samples in glass containers (9.9 log_10_ CFU/mL) showed higher final LAB count if compared to those fermented in clay pots (9.6 log_10_ CFU/mL).

### 3.2. Effect of Fermentation Container on Apparent Viscosity and Whiteness Index

**Figure 2:**
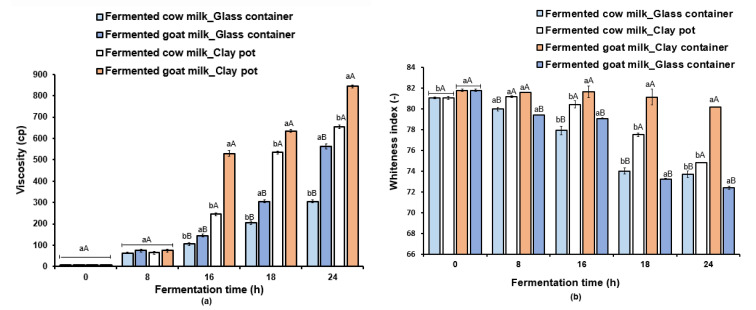
Changes in viscosity (**a**) and whiteness index (**b**) variations during cow and goat milk fermentation. ^a,b^ for each type of milk, different letters indicate significant differences using the Tukey test (*p* < 0.05). ^A,B^ for each type of container used for milk fermentation, capital letters describe a significant difference using the Tukey test (*p* < 0.05).

Figure 2 depicts the variations in the viscosity and whiteness index of cow milk and goat milk during fermentation. There were no significant differences (*p* > 0.05) in apparent viscosity values during the first 8 h of fermentation (Figure 2a). The viscosity increased depending on the type of milk and container used. The apparent viscosity was higher in clay pot for goat milk, with a mean value of 845 ± 7.1 cP reached within 24 h.

Figure 2b shows the whiteness index variation according to the type of milk and container used. Fermented milk obtained using clay pots showed high whiteness index values for both goat and cow milk samples, and higher values were noted for goat milk at different sampling times. After 8 h of milk fermentation, the difference between samples was mainly attributed to the milk container, and from 8 to 24 h, the interaction term milk × container was found significant for apparent viscosity (Figure 2a).

### 3.3. Effect of Type of Milk and Container on Nutritional Profile

Table 1 illustrates the proximate composition of raw and fermented milk samples. Compared to the composition of cow milk, goat milk was characterized by higher fat (3.5 ± 0.2 g/100 g), total solids (13.1 ± 0.9 g/100 g), and ash (0.878 ± 0.002 g/100 g) contents. Protein, fat, total solids, ash, and lactose contents decreased significantly (*p* < 0.05) after fermentation for goat milk. Higher values of ash (0.798 ± 0.003 g/100 g) and lactose (3.7 ± 0.1 g/100 g) were obtained in fermented goat milk in the clay pot. A significant decrease (*p* < 0.05) was also recorded for protein, total solids, and lactose contents in fermented cow milk. Compared to *FCM_Glass container*, *FCM_Clay pot* corresponds to higher protein (3.1 ± 0.1 g/100 g), total solids (11.5 ± 0.4 g/100 g), ash (0.79 ± 0.02 g/100 g), and lactose (3.4 ± 0.1 g/100 g) contents.

### 3.4. Influence of Type of Milk and Container Used for Milk Fermentation on Fatty Acids and Phenolic Compounds

Table 2 summarizes the fatty acid composition of the different fermented milk samples. In total, 17 fatty acids were identified in raw and fermented cow and goat milk, whereas myristoleic acid was detected only in raw and fermented cow milk. The obtained results showed that palmitic, capric, stearic, and oleic acids were the main detected fatty acids. Higher percentages of caproic, caprylic, and capric acid were found in raw and fermented goat milk than those measured in cow milk. Oleic acid was found to be higher in cow milk than in goat milk.

The pairwise comparison revealed significant differences in butanoic, caprylic, capric, lauric, stearic, oleic, linoleic, and α-linolenic acid contents according to the container type. Higher contents of butanoic, caprylic, capric, and lauric acids were recorded in the glass container whereas higher amounts of linoleic, and α-linolenic were recorded in the clay container.

Among the principal phenolic compounds in raw and fermented milks, eight compounds were identified, with quinic acid being the major compound in raw and fermented milk (Table 3). Fermented goat milk recovered higher values of quinic acid if compared to cow milk (42.67 ± 0.1 mg/L extract for fermented goat milk in a clay pot and 42.89 ± 0.1 mg/L extract for fermented goat milk in a glass container). Apigenin and cirsiliol were also identified in raw and fermented milk samples at lower concentrations. Salviolinic acid was present only in raw goat milk. Rutin and quercetin were detected in goat milk fermented in the clay pot, whereas luteolin was found in goat milk fermented in the glass container.

### 3.5. Microbial Profiles of Goat and Cow Milk Fermented in Glass and Clay Containers

The analysis of 16s rRNA by NGS produced an average of 68.4 × 10^3^ read pairs per sample (min: 47.1 × 10^3^; max:129.7 × 10^3^) that were reduced uniformly to about 64–71% after the denoising procedure (Supplementary Table S3), indicating no differences in terms of sequencing yield among the samples. Analysis of the taxonomic profiles revealed a significant variation between the samples.

**Figure 3:**
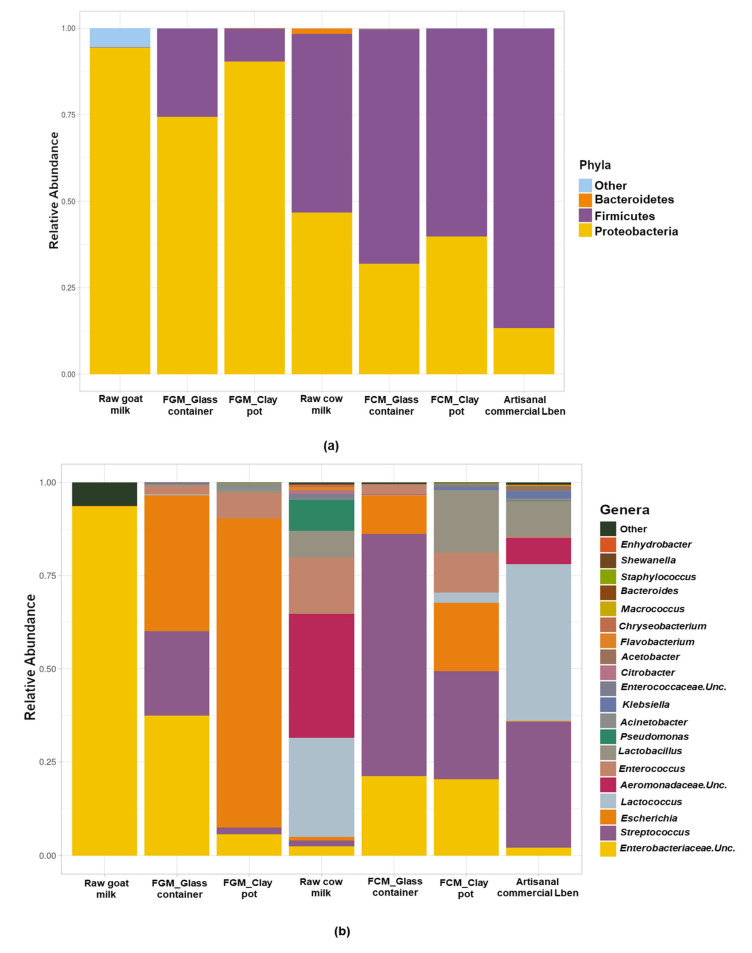
Microbiota composition of raw and fermented milk samples and artisanal fermented Lben from local producer represented by relative abundance of phyla (**a**) and genera (**b**) of the abundant bacterial operational taxonomic units (OTUs). FGM: Fermented Goat Milk; FCM: Fermented Cow Milk; Different colors in bars described different OTUs.

The distribution of phyla and genera in fermented goat and cow samples is shown in Figure 3. *Proteobacteria* was found to be the most prevalent phylum in the goat milk samples according to the sequencing results, whereas *Firmicutes* dominated the fermented cow milk and commercial milk (Figure 3a). At the genus level, *Streptococcus* spp., *Escherichia* spp., *Lactococcus* spp., *Enterococcus* spp., and *Lactobacillus* spp. were identified as the most abundant taxa, with a low abundance of other genera such as *Enterococcus* and *Aeromonadaceae* (Figure 3b).

Raw goat milk was the richest of the *Enterobacteriaceae* family, whereas raw cow milk had more varied microbiota. The genera *Escherichia* spp. and *Streptococcus* spp. were found in fermented milk; in particular, the first genus was most prevalent in fermented goat milk, whereas the latter was mostly present in cow products (Figure 3b). Commercial artisanal Lben has a more diverse bacterial microbiota, with *Lactococcus* spp. and *Streptococcus* spp. being the two most prevalent families.

**Figure 4:**
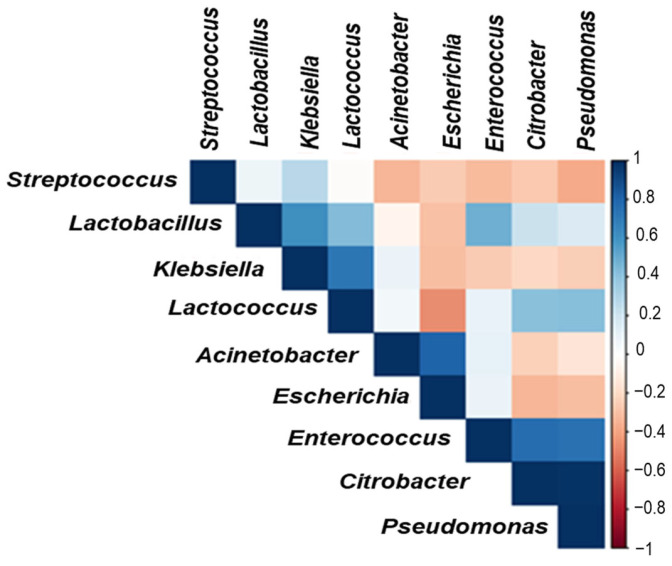
Correlation analysis between major genera identified in the raw and spontaneously fermented cow milk and goat milk. Color scale indicates the correlation coefficient ranging from 1 (blue) to −1 (red).

Correlation analysis among the major genera in Figure 4 illustrates the inter-genera competition. Interestingly, our data revealed that *Escherichia* spp. abundance negatively correlated with many genera, including *Lactobacillus* spp. and *Lactococcus* spp. *Streptococcus* spp., *Lactococcus* spp., and *Lactobacillus* spp. were positively correlated.

## 4. Discussion

This study investigated the effects of milk type (goat and cow) and fermentation vessels (glass and clay pot) on the microbiota diversity, fatty acids, phenolic profiles, and the main nutritional and physicochemical properties of spontaneously fermented milk.

The fermentation vessels significantly influenced the microbial and nutrient composition of fermented dairy products. For some traditional products, the use of artisanal material for processing or storage is mandatory and protected by local regulations for products with protected denomination of origin (PDO) [28]. Clay pot is used to store or prepare cultured milk in rural areas [29,30]. It is preferred because it absorbs the excess of whey and allows specific sensory properties, mainly in terms of flavor and texture. Moreover, clay pots are characterized as permeable materials to moisture and gases, and do not react with foods compared to plastic containers which are still used in many countries [31].

In this study, statistical results for milk acidification tendency represented by pH and acidity curves provided insight into the significant effects of milk type and fermentation container. As expected, for milk acidification, there was a significant difference depending on the type of milk in the first 3 h of fermentation, while the influence of the container was noticed later, after a lag time of 2 to 6 h. The use of a glass container resulted in higher acidification rates for both types of milk, which could be attributed to the variations in temperature transfer depending on the composition and physical characteristics of the vessel material. This result is in agreement with the reported literature considering the clay pot as cooling devices for food preservation in hot and dry climates according to Date [32]. Additionally, the interaction term type of milk × container has a significant effect on pH and acidity kinetics (*p* < 0.05). Groenenboom et al. [33] reported similar trends for buckets and calabashes containers used in *Mabisia* traditional fermented cow milk in Zambia. They found that the fermentation activity had a lag time depending on the re-use of cleaned containers and the back-slopping method, with a final pH below 5 within 24 h of fermentation similarly to our results.

The total LAB count found in this study is in the similar range reported in previous studies dealing with growth trends in traditional fermented milk [22]. The use of clay pot resulted in a lower LAB count than the glass container due to the cooling material’s characteristics, as mentioned above. This also might be a result of the adherence activity of lactic bacteria from raw milk naturally to the container’s inner surface [11]. The re-use of the same container for milk fermentation can speed up the growth due to the development of LAB biofilm on the surface [28], while the re-use of the same container for milk fermentation can affect the quality and the safety of the obtained product.

Viscosity and color are among main organoleptic characteristics that distinguish fermented milk beverages [23]. Few studies have investigated the effect of fermentation vessels on the variation of apparent viscosity and color parameters of fermented goat milk. A study conducted by Priyashantha et al. [31] on Meekiri, a traditional dairy product made in clay pot, showed that the fermented milk was characterized by a high viscosity and creamy and thick mouthfeel attributes. Moonga et al. [11] for Mabisi also reported similar results, describing that higher viscosity was also correlated to the fermenting bacteria consortia [33]. The color of fermented dairy products changes from light yellow to white depending on the milk’s composition and the fermentation environment [34]. The whiteness indices of goat and cow milk samples decreased during the fermentation process. Goat milk fermented in a clay pot showed higher whiteness index values than the other samples. Similar to our findings, Vargas et al. [35] found that goat milk yogurt had a higher whiteness index than cow milk yogurt. This difference was attributed to the formed gel’s opacity, which was caused by the casein ratio and protein aggregation, the absence of β-carotene in goat milk, and the presence of small fat globules [36]. On the other hand, acidification rate may promote the variation of the color index due to substances produced by proteolysis and lipolysis reactions, which are accelerated or slowed during fermentation according to used vessels [37]. The combination mixture of microorganisms influenced the color profile of fermented milk, mainly lactic bacteria [38]. Organic compounds derived from lactic bacteria, such as reuterin produced by *Lb. reuteri*, could also influence the color of fermented milk. It was reported that fermented milk products without reuterin had higher L* values than fermented milk products with reuterin. However, fermented milk products with reuterin displayed higher a* and b* values [39].

The nutritional composition of fermented milk is dependent on the type of milk and fermentation container. Overall, the obtained results are in agreement with those of Chileshe et al. [10], Samet-Bali et al. [40], and Agyei et al. [41].

Milk and fermented dairy products contain bioactive molecules, with proteins and fatty acids being the most prevalent and having nutritional and technological interest [42]. The fatty acid profiles of raw and fermented milk samples significantly varied, but overall, the type of milk was the main determining factor. Fermented goat milk contained large amounts of short-chain fatty acids, which can be explained by the fact that goat milk contains more capric acid than cow milk. On the other hand, cow fermented milk contained more long-chain fatty acids, primarily oleic acid. Lucatto et al. [43] reported that the polyunsaturated and short-chain fatty acids were higher in goat milk yoghurt than those in cow milk yoghurt.

The majority of phenolic compounds in ruminant milk come from animal feed, although some may be produced through the catabolism of amino acids [44]. As previously reported by Jordán et al. [45], two classes of phenolic compounds, lipophilic and hydrophilic, are transferred to milk depending on their molecular weight to cross lipid membranes. This study identified both lipophilic and hydrophilic compounds in raw and fermented milk, with quinic acid being the most prevalent chlorogenic acid derivative. Previous research suggested that higher concentrations of chlorogenic acids result from the metabolism of tannic acid and other polyphenolic compounds [46]. Phenolic compounds in milk and fermented foods are crucial for auto-bio-preservation and sensory qualities, particularly food flavor and human nutrition [46]. Additionally, phenolic compounds are bio-transformed into derivatives, and may have prebiotic properties that promote the growth of LAB [47].

Significant variations in microbial communities were identified among samples. *Escherichia* spp. was the subdominant genera from the *Enterobacteriaceae* family. No other food spoilage organisms such as *Staphylococcus* spp. and *Listeria* spp. were detected. Previous studies on traditional dairy products made from unpasteurized milk [46] reported the presence of pathogenic bacteria due to the quality of raw milk and uncontrolled conditions of milk fermentation, such as handling procedures and hygiene deficiencies in the equipment [48]. Fermented cow milk samples showed a lower level of *Escherichia* spp. and a greater diversity of microbiota with *Streptococcus* spp. genera predominating. A higher distribution of *Escherichia* spp. was found when clay pot was used for both goat and cow milks compared to the glass container. This may be explained by the interactions between biotic and abiotic factors. Indeed, the fermenting substrate is supported by the dominance of *Enterobacteriaceae* in raw goat milk and the acidification progression using clay pot. According to Groenenboom et al. [33], the use of fermentation vessels with a larger opening, such as the clay pot used in this study, resulted in the transfer of environmental bacteria into the fermentation media both before and during fermentation. Furthermore, pathogenic bacteria may replace LAB due to the clay pot interior’s rough surface and to its low heat conductivity.

In this work dealing with goat and cow fermented milk, the main LAB genera identified were *Lactobacillus* spp., *Lactococcus* spp., and *Streptococcus* spp. The latter is the most prevalent, with an increasing trend during fermentation as reported for the Chhurpi and Churkam products [49]. The increase in *Streptococcus* levels from raw to fermented milk could be correlated to the total LAB growth (Figure 1c) in cow milk fermented in the glass container, showing a higher rate of growth.

A comparison of the microbial community in fermented cow and goat milk made at a laboratory scale, and that of traditional commercial fermented milk Lben, allows us to underline potential environmental contamination factors. The presence of *Streptococcus* spp., *Lactococcus* spp. and *Lactobacillus* spp. was reported by sequencing for the first time in fermented milk. According to the microbiota composition, *Streptococcus* spp. and *Lactococcus* spp. were the most abundant, and were also detected in all samples with low abundance. *Enterobacteria* were found in low abundance compared to the other fermented milk samples. This variability among the analyzed samples could be attributed to the variability in microbial diversity in raw milk and the impact of process conditions as previously reported for similar dairy products [33].

An interesting strong negative correlation was found between spoilage and lactic acid bacteria, specifically for *Escherichia* spp. The pathogenic bacterial species showed a weak correlation between them, while the LAB showed the strongest positive correlation among the identified bacteria. Similar correlations were found by Secchi et al. [50] and can be attributed to the greater diversity of the entire bacterial community.

It is interesting to note that the use of clay pot produced fermented milk with a particular fatty acid profile, thick viscosity, and a higher whiteness index. However, the final bacterial composition showed that the clay container developed more spoilage bacteria that were already present in raw milk than when using glass containers. *Escherichia* spp. were most abundant in fermented milks using the clay pot compared to the glass container. Previous works [19,51] recommended the use of raw milk with high microbiological quality and clay containers with inner boards laced with lactic bacteria biofilms to inhibit pathogenic bacteria and to ensure the particular sensory properties of products. Safety precautions such as the use of pasteurized milk, starter cultures, and a safe and controlled environment are recommended. Good practices and safety control measurements remain more appropriate for ensuring the safety of specific traditional fermented dairy products with protected denominations.

## 5. Conclusions

Our findings demonstrated a significant influence of the fermentation container and type of milk on pH, acidity, proximate composition, and viscosity of fermented milk. The whiteness index, fatty acids, and phenolic compounds were mainly affected by milk type. Microbial communities of fermented milk and their variations depending on the type of milk (goat and cow) and used container for fermentation (glass and clay) were also investigated. Both type of milk and container had a significant effect on bacterial predominance in microbial communities. These findings showed that metataxonomic sequencing analysis is an effective tool that allows determination of microbial diversity in traditional fermented dairy products and could be used to control their quality.

## Figures and Tables

**Table 1:** Proximate analysis of raw and fermented milk samples.

Milk Samples	Aw (-)	Lactose (g/100 g)	Ash (g/100 g)	Total Solids (g/100 g)	Fat (g/100 g)	Protein (g/100 g)
Raw goat milk	0.986 ± 0.001 ^a^	4.5 ± 0.1 ^a^	0.878 ± 0.002 ^a^	13.1 ± 0.9 ^a^	3.5 ± 0.2 ^a^	3.3 ± 0.1 ^a^
*FGM_Clay pot*	0.980 ± 0.004 ^b^	3.7 ± 0.1 ^b^	0.798 ± 0.003 ^b^	12.0 ± 0.8 ^ab^	3.3 ± 0.3 ^ab^	3.1 ± 0.2 ^ab^
*FGM_Glass container*	0.985 ± 0.001 ^a^	3.3 ± 0.1 ^cd^	0.700 ± 0.001 ^c^	11.5 ± 0.3 ^ab^	2.8 ± 0.3 ^b^	3.1 ± 0.1 ^ab^
Raw cow milk	0.980 ± 0.004 ^b^	4.2 ± 0.1 ^a^	0.82 ± 0.01 ^b^	12.8 ± 0.6 ^ab^	3.3 ± 0.2 ^ab^	3.2 ± 0.2 ^a^
*FCM_Clay pot*	0.973 ± 0.001 ^c^	3.4 ± 0.1 ^bc^	0.79 ± 0.02 ^b^	11.5 ± 0.4 ^ab^	3.1 ± 0.2 ^ab^	3.1 ± 0.1 ^ab^
*FCM_Glass container*	0.980 ± 0.003 ^b^	3.1 ± 0.1 ^d^	0.71 ± 0.02 ^c^	11.4 ± 0.3 ^b^	3.1 ± 0.2 ^ab^	2.8 ± 0.1 ^b^

FGM: Fermented Goat Milk; FCM: Fermented Cow Milk; S: significant; NS: not significant; Mean values with different letters in the same column are significantly different *p* < 0.05.

**Table 2:** Fatty acids composition (% of total fatty acids) of raw and fermented milk in clay pot and glass container.

Fatty Acids (%)	RGM	FGM_Clay Pot	FGM_Glass Container	RCM	FCM_Clay Pot	FCM_Glass Container
***Saturated fatty acids***						
*Butanoic acid (C4:0)*	2.40 ± 0.03	1.92 ± 0.2 ^ab^	2.4 ± 0.1 ^a^	2.3 ± 0.2	1.45 ± 0.02 ^b^	2.1 ± 0.1 ^a^
*Caproic acid (C6:0)*	2.72 ± 0.02	2.05 ± 0.1 ^ab^	2.9 ± 0.1 ^a^	1.4 ± 0.1	1.3 ± 0.1 ^b^	1.2 ± 0.1 ^b^
*Caprylic acid (C8:0)*	3.17 ± 0.01	2.4 ± 0.1 ^b^	3.5 ± 0.2 ^a^	0.864 ± 0.005	0.78 ± 0.01 ^c^	0.81 ± 0.02 ^c^
*Capric acid (C10:0)*	11.97 ± 0.03	7.5 ± 0.4 ^b^	11.8 ± 0.2 ^a^	2.14 ± 0.01	2.002 ± 0.004 ^c^	2.01 ± 0.01 ^c^
*Lauric acid (C12:0)*	4.3 ± 0.7	3.47 ± 0.03 ^b^	4.33 ± 0.03 ^a^	2.85 ± 0.03	2.71 ± 0.02 ^c^	2.68 ± 0.01 ^c^
*Myristic acid (C14:0)*	9.2 ± 0.1	10.2 ± 0.2 ^a^	10.20 ± 0.01 ^a^	10.40 ± 0.01	10.4 ± 0.2 ^a^	10.3 ± 0.2 ^a^
*Pentadecanoic acid (C15:0)*	1.01 ± 0.02	1.13 ± 0.01 ^a^	0.9 ± 0.1 ^a^	1.41 ± 0.04	1.3 ± 0.1 ^a^	1.34 ± 0.04 ^a^
*Palmitic acid (C16:0)*	26.9 ± 0.8	29.15 ± 0.05 ^a^	30.7 ± 0.2 ^a^	29.03 ± 0.3	30.31 ± 0.03 ^a^	30.3 ± 1.2 ^a^
*Heptadecanoic acid (C17:0)*	0.66 ± 0.01	0.92 ± 0.04 ^a^	0.5 ± 0.1 ^a^	0.73 ± 0.01	0.6 ± 0.1 ^a^	0.62 ± 0.04
*Stearic acid (C18:0)*	11.9 ± 0.1	14.1 ± 0.3 ^a^	11.4 ± 0.2 ^b^	9.7 ± 0.2	9.4 ± 0.2 ^b^	9.5 ± 0.1 ^b^
***Arachidic acid (C20:0)***	0.70 ± 0.01	0.3 ± 0.1 ^a^	0.27 ± 0.03 ^a^	2.23 ± 0.01	0.15 ± 0.01 ^a^	0.13 ± 0.01
***Mono-saturated fatty acids***						
*Cis-9-hexadecenoic acid (C16:1)*	0.5 ± 0.01	0.6 ± 0.04 ^b^	0.30 ± 0.01 ^c^	2.33 ± 0.01	1.94 ± 0.04 ^a^	1.7 ± 0.2 ^a^
*Cis-10- heptadecenoic acid (C17:1)*	0.2 ± 0.04	0.3 ± 0.04 ^a^	nd	0.3 ± 0.1	0.29 ± 0.03 ^a^	0.34 ± 0.03 ^a^
*Oleic acid (C18:1)*	18.97 ± 0.1	25.2 ± 0.1 ^b^	20.7 ± 0.01 ^c^	30.0 ± 0.1	31.3 ± 0.3 ^a^	31.2 ± 0.9 ^a^
*Myristoleic acid (C14:1)*	nd	nd	nd	1.30 ± 0.02	1.20 ± 0.04 ^a^	1.22 ± 0.02 ^a^
***Polyunsaturated fatty acids***						
*Linoleic acid (C18:2)*	2.14 ± 0.01	3.4 ± 0.2 ^a^	1.8 ± 0.1 ^b^	4.6 ± 0.1	4.3 ± 0.04 ^a^	4.2 ± 0.1 ^a^
*Cis-9,12,15-octadecatrienoic (α-linolenic) (C18:3)*	0.21 ± 0.03	0.62 ± 0.02 ^a^	0.22 ± 0.01 ^b^	0.25 ± 0.02	0.25 ± 0.01 ^b^	0.21 ± 0.01 ^b^

RGM: Raw Goat Milk; RCM: Raw Cow Milk; FGM: Fermented Goat Milk; FCM: Fermented Cow Milk; nd: not detected; Mean values with different letters in the same column are significantly different *p* < 0.05.

**Table 3:** LC-MS analysis of phenolic compounds identified in raw and fermented milk.

Peak	Retention Time (min)	Compound	Chemical Formula	[M-H] *m*/*z*	Concentration (mg/L_extract_)					
					RGM	FGM_Clay Pot	FGM_ Glass Container	RCM	FCM_ Clay Pot	FCM_ Glass Container
1	2.269	quinic acid	C_7_H_12_O_6_	191	29.59 ± 0.10	42.67 ± 0.10	42.89 ± 0.10	25.2 ± 0.1	34.03 ± 0.1	26.2 ± 0.1
2	27.734	Rutin	C_27_H_30_O_16_	609	nd	0.003 ± 0.003	nd	nd	nd	nd
3	33.292	Salviolinic acid	C_36_H_30_O_1_	717	1.184 ± 0.001	nd	nd	nd	nd	nd
4	35.502	quercetin	C_15_H_10_O_7_	301	nd	0.012 ± 0.002	nd	nd	nd	nd
5	36.915	Naringenin	C_15_H_12_O_5_	271	0.007 ± 0.003	nd	nd	nd	nd	nd
6	38.107	Luteolin	C_21_H_20_O_11_	285	nd	nd	0.038 ± 0.001	nd	nd	nd
7	38.816	Apigenin	C_15_H_10_O_5_	269	0.018 ± 0.001	0.035 ± 0.001	0.003 ± 0.001	0.058 ± 0.001	0.043 ± 0.001	0.029 ± 0.001
8	39.015	Cirsiliol	C_17_H_14_O_7_	329	0.010 ± 0.003	0.012 ± 0.003	0.018 ± 0.003	0.015 ± 0.003	0.017 ± 0.003	0.009 ± 0.003

RGM: Raw Goat Milk; RCM: Raw Cow Milk; FGM: Fermented Goat Milk; FCM: Fermented Cow Milk; nd: not detected.

## Data Availability

16s sequencing data are available at SRA-NCBI portal under the bioproject PRJNA954593.

## Supplementary Materials

The following supporting information can be downloaded at: https://www.mdpi.com/article/10.3390/foods12091836/s1, Table S1: Groupwise summary statistics for pH and acidity; Table S2: Groupwise summary statistics for lactic bacteria count; Table S3: Paired ends data of sequences for metataxonomic analysis.
