# Supplementary material for: Influence of Fermentation Container Type on Chemical and Microbiological Parameters of Spontaneously Fermented Cow and Goat Milk
Source: Foods. 2023 Apr 28;12(9):1836. doi: 10.3390/foods12091836 (PMC10177932; doi:10.3390/foods12091836)
Supplement: Supplementary file 1 [file foods-12-01836-s001.zip › foods-2319564-supplementary.pdf]

Supplementary Table S1. Groupwise summary statistics for pH and acidity

Variables	factors	Fermentation time (h)												
		0	2	4	6	8	10	12	14	16	18	20	22	24
pH	Clay pot	6.66 ^{a}	6.55 ^{a}	6.51 ^{a}	6.46 ^{a}	6.34 ^{a}	5.84 ^{a}	5.52 ^{a}	5.16 ^{a}	4.93 ^{a}	4.73 ^{a}	4.61 ^{a}	4.53 ^{a}	4.44 ^{a}
	Glass container	6.66 ^{a}	6.56 ^{a}	6.51 ^{a}	6.37 ^{b}	5.95 ^{b}	5.35 ^{b}	4.88 ^{b}	4.61 ^{b}	4.56 ^{b}	4.51 ^{b}	4.45 ^{b}	4.41 ^{b}	4.36 ^{b}
	Pr > F (container)	1.000	0.176	0.347	< 0.0001	< 0.0001	< 0.0001	< 0.0001	< 0.0001	< 0.0001	< 0.0001	< 0.0001	< 0.0001	< 0.0001
	Significant	No	No	No	Yes	Yes	Yes	Yes	Yes	Yes	Yes	Yes	Yes	Yes
	Fermented cow milk	6.71 ^{a}	6.63 ^{a}	6.59 ^{a}	6.50 ^{a}	6.39 ^{a}	5.89 ^{a}	5.30 ^{a}	4.78 ^{b}	4.69 ^{b}	4.63 ^{a}	4.53 ^{a}	4.46 ^{a}	4.41 ^{a}
	Fermented goat milk	6.61 ^{b}	6.48 ^{b}	6.43 ^{b}	6.33 ^{b}	5.90 ^{b}	5.30 ^{b}	5.11 ^{b}	4.99 ^{a}	4.79 ^{a}	4.61 ^{b}	4.52 ^{a}	4.47 ^{a}	4.39 ^{b}
	Pr > F (milk)	< 0.0001	< 0.0001	< 0.0001	< 0.0001	< 0.0001	< 0.0001	< 0.0001	< 0.0001	< 0.0001	0.035	0.195	0.392	0.013
	Significant	Yes	Yes	Yes	Yes	Yes	Yes	Yes	Yes	Yes	Yes	No	No	Yes
	Clay pot*Fermented cow milk	6.72 ^{a}	6.62 ^{a}	6.60 ^{a}	6.54 ^{a}	6.44 ^{a}	5.95 ^{a}	5.51 ^{b}	4.99 ^{b}	4.85 ^{b}	4.75 ^{a}	4.58 b	4.51 ^{a}	4.45 ^{a}
	Clay pot*Fermented goat milk	6.61 ^{b}	6.48 ^{b}	6.43 ^{b}	6.38 ^{c}	6.34 ^{b}	5.83 ^{b}	5.53 ^{a}	5.34 ^{a}	5.01 ^{a}	4.70 ^{b}	4.63 a	4.55 ^{a}	4.44 ^{a}
	Glass container*Fermented cow milk	6.72 ^{a}	6.64 ^{a}	6.58 ^{a}	6.46 ^{b}	6. 24 ^{c}	5.73 ^{c}	5.08 ^{c}	4.57 ^{d}	4.53 ^{c}	4.50 ^{c}	4.47 c	4.42 ^{b}	4.37 ^{b}
	Glass container*Fermented goat milk	6.61 ^{b}	6.48 ^{b}	6.44 ^{b}	6.28 ^{d}	5.56 ^{d}	4.87 ^{d}	4.68 ^{d}	4.65 ^{c}	4.59 ^{c}	4.52 ^{c}	4.42 d	4.40 ^{b}	4.35 ^{b}
	Pr > F (container*milk)	1.000	0.373	0.081	0.360	< 0.0001	< 0.0001	< 0.0001	< 0.0001	0.004	0.001	< 0.0001	0.042	0.320
	Significant	No	No	No	No	Yes	Yes	Yes<	Yes	Yes	Yes	Yes	Yes	No
Acidity	Glass container	1.65 ^{a}	1.67 ^{a}	1.69 ^{a}	1.84 ^{a}	2.10 ^{a}	2.82 ^{a}	3.33 ^{a}	5.07 ^{a}	5.99 ^{a}	7.54 ^{a}	7.86 ^{a}	8.08 ^{a}	8.34 ^{a}
	Clay pot	1.65 ^{a}	1.66 ^{a}	1.68 ^{a}	1.73 ^{b}	1.85 ^{b}	2.04 ^{b}	2.23 ^{b}	3.48 ^{b}	4.86 ^{b}	6.59 ^{b}	7.38 ^{b}	7.66 ^{b}	7.87 ^{b}
	Pr > F (container)	0.842	0.216	0.388	0.001	0.000	< 0.0001	< 0.0001	< 0.0001	< 0.0001	< 0.0001	< 0.0001	< 0.0001	0.000
	Significant	No	No	No	Yes	Yes	Yes	Yes	Yes	Yes	Yes	Yes	Yes	Yes
	Fermented cow milk	1.68 ^{a}	1.71 ^{a}	1.73 ^{a}	1.85 ^{a}	2.12 ^{a}	2.48 ^{a}	2.78 ^{a}	3.33 ^{b}	4.91 ^{b}	6.95 ^{b}	7.86 ^{a}	8.06 ^{a}	8.32 ^{a}
	Fermented goat milk	1.62 ^{b}	1.61 ^{b}	1.64 ^{b}	1.72 ^{b}	1.82 ^{b}	2.38 ^{a}	2.78 ^{a}	5.23 ^{a}	5.94 ^{a}	7.18 ^{a}	7.37 ^{b}	7.68 ^{b}	7.88 ^{b}
	Pr > F (milk)	0.004	< 0.0001	< 0.0001	0.000	0.000	0.134	0.929	< 0.0001	< 0.0001	0.001	< 0.0001	< 0.0001	0.000
	Significant	Yes	Yes	Yes	Yes	Yes	No	No	Yes	Yes	Yes	Yes	Yes	Yes
	Glass container*Fermented cow milk	1.68 ^{a}	1.72 ^{a}	1.74 ^{a}	1.95 ^{a}	2.42 ^{a}	3.03 ^{a}	3.67 ^{a}	4.20 ^{c}	5.35 ^{b}	7.55 ^{a}	8.10 ^{a}	8.30 ^{a}	8.65 ^{a}
	Glass container*Fermented goat milk	1.62 ^{a}	1.61 ^{b}	1.64 ^{b}	1.75 ^{b}	1.77 ^{b}	2.61 ^{b}	3.00 ^{b}	5.95 ^{a}	6.63 ^{a}	7.52 ^{a}	7.62 ^{b}	7.86 ^{b}	8.03 ^{b}
	Clay pot*Fermented cow milk	1.68 ^{a}	1.70 ^{a}	1.72 ^{a}	1.72 ^{b}	1.82 ^{b}	1.93 ^{c}	1.90 ^{d}	2.45 ^{d}	4.47 ^{c}	6.35 ^{c}	7.61 ^{b}	7.81 ^{c}	8.00 ^{b}
	Clay pot*Fermented goat milk	1.62 ^{a}	1.61 ^{b}	1.64 ^{b}	1.71 ^{b}	1.87 ^{b}	2.15 ^{c}	2.55 ^{c}	4.51 ^{b}	5.25 ^{b}	6.83 ^{b}	7.13 ^{c}	7.51 ^{d}	7.74 ^{b}
	Pr > F (container*milk)	0.842	0.274	0.388	0.003	< 0.0001	0.001	< 0.0001	0.035	0.001	0.001	0.976	< 0.0001	0.024
	Significant	No	No	No	Yes	Yes	Yes	Yes	Yes	Yes	Yes	No	Yes	Yes

Supplementary Table S2. Groupwise summary statistics for lactic bacteria count

Factors	Fermentation time (h)								
	0	3	6	9	12	15	18	21	24
Glass container	6.553 a	6.511 a	7.948 a	8.146 a	8.055 a	9.414 a	9.625 a	9.999 a	9.895 a
Clay pot	6.553 a	6.564 a	6.794 b	6.806 b	7.925 a	8.851 b	9.018 b	9.570 b	9.624 b
Pr > F(container)	1.000	0.390	< 0.0001	< 0.0001	0.169	< 0.0001	< 0.0001	0.002	0.003
Significant	No	No	Yes	Yes	No	Yes	Yes	Yes	Yes
Fermented cow milk	6.770 a	6.722 a	7.429 a	7.554 a	8.206 a	9.283 a	9.637 a	9.736 a	9.825 a
Fermented goat milk	6.335 b	6.353 b	7.312 a	7.397 b	7.774 b	8.982 b	9.006 b	9.833 a	9.695 a
Pr > F(milk)	0.001	0.000	0.172	0.026	0.001	< 0.0001	< 0.0001	0.319	0.075
Significant	Yes	Yes	No	Yes	Yes	Yes	Yes	No	No
Fermented cow milk*glass	6.770 a	6.782 a	8.133 a	8.248 a	8.289 a	9.726 a	10.209 a	10.015 a	9.980 a
Fermented goat milk*glass container	6.335 b	6.240 c	7.763 b	8.044 a	7.821 bc	9.102 b	9.040 b	9.983 a	9.811 ab
Fermented cow milk*clay pot	6.770 a	6.663 ab	6.726 c	6.861 b	8.123 ab	8.841 c	9.064 b	9.457 b	9.670 b
Fermented goat milk*clay pot	6.335 b	6.466 bc	6.862 c	6.751 b	7.727 c	8.862 c	8.972 b	9.684 ab	9.579 b
Pr > F(milk*container)	1.000	0.019	0.012	0.432	0.687	< 0.0001	< 0.0001	0.194	0.563
Significant	No	Yes	Yes	No	No	Yes	Yes	No	No

Supplementary Table S3. Paired ends data of sequences for metataxonomic analysis

Source	Milk type	Container	#raw seqeunces	#denoised sequences	denoising (%)
goat	raw	n.a.	129742	91775	70.74
goat	fermented	glass	62838	43336	68.96
goat	fermented	clay	53934	37566	69.65
cow	raw	n.a.	53884	37184	69.01
cow	fermented	glass	47071	33560	71.3
cow	fermented	clay	73209	46642	63.71
cow	fermented	Lben from local producer	58024	37385	64.43
